# The Effect of Fibrin Glue on the Intensity of Colonic Anastomosis in the Presence and Absence of Peritonitis: An Experimental Randomized Controlled Trial on Rats

**DOI:** 10.1155/2013/521413

**Published:** 2013-01-21

**Authors:** Metin Senol, Mehmet M. Altintas, Ayhan Cevık, Yunus E. Altuntas, Nagehan O. Barisik, Nejdet Bildik, Mustafa Oncel

**Affiliations:** ^1^Department of General Surgery, Nevsehir State Hospital, 50000 Nevsehir, Turkey; ^2^Department of General Surgery, Kartal Education and Research Hospital, Istanbul, Turkey; ^3^Department of Pathology, Kartal Education and Research Hospital, Istanbul, Turkey

## Abstract

*Aim*. Anastomotic leakage after colon anastomosis is the most frequent and most feared complication with its highest mortality rate. In this study, we aimed to expose the impact of performing fibrin glue on sutured colocolic anastomosis, in the presence of experimental peritonitis, on anastomosis safety. *Method*. In this experimental study, the rats were divided into two groups as control group (Groups 1 and 3) and experimental group (Groups 2 and 4). They were also divided as clean abdomen (Groups 1 and 2) and infected abdomen (3 and 4) groups. Full-thickness incisions were made on the proximal colon of both groups of rats. The control group's anastomoses were conducted only with sutures, whereas in experimental group, fibrin glue was applied over the sutures. The samples were taken on the 10th day. *Results*. Highest values for average levels of hydroxyproline in the tissues and anastomotic bursting pressures were detected when fibrin glue was applied on sutured anastomosis in clean abdomen. In the histopathological staging performed in line with Ehrlich-Hunt model, lowest values were detected during the presence of peritonitis. *Conclusion*. As a result, it has been established that the use of fibrin glue over sutured colocolic anastomosis, both in clean abdomen and in the presence of peritonitis, had increased anastomosis safety.

## 1. Introduction

In colorectal surgery, intestinal anastomoses are being performed in lots of intestinal pathologies, mainly, malign colon tumors. The complications observed after colon anastomoses are anastomotic leakage, fistula, hemorrhage, and anastomotic stricture. The most common of such complications is anastomotic leakage with its highest morbidity and mortality rates [1]. There are variety of factors besides the surgeon's lack of experience and skill which can lead to anastomotic complications such as patient's advanced age, presence of additional diseases such as diabetes mellitus, weight loss, hypotension, urgent operations, and infections [2]. 

Both in elective and in urgent colon operations, primary anastomosis is being avoided in case of infected abdomen and multistep procedures are preferred [3, 4]. The reason for this is the deterioration of wound recovery in a contaminated environment and high risk of anastomotic leakage [5]. It has to be remembered that performing ostomy instead of anastomosis may cause many complications, loss of work power, and increase in cost and thus burden additional morbidity on patients [4, 6]. Therefore, anastomosis safety has been one of the most studied topics in colorectal surgery. 

To prevent anastomotic leakage, various techniques of anastomosis are recommended as well as trying various preoperative methods such as antibiotic prophylaxis, preoperative intestinal preparation, and fecal diversion with proximal ostomies [6]. Applying fibrin glue on anastomosis is one of these methods too [7]. As a matter of fact, it is stated that fibrin glue will be sufficient by itself for the colon anastomosis and provide anastomosis safety equating the one with sutures [8]. 

Fibrin glue used in this study (Tisseel VH) has been licensed as an auxiliary treatment for homeostasis firstly in cardiopulmonary bypass surgery, for treatment of spleen damages in blunt or penetrating abdominal trauma, when suture, ligation, or cauterization applied to control bleeding during surgical procedures is insufficient or impossible. It consists of four flacons as fibrinogen, aprotinin, trombone, and calcium. When these are brought together, fibrin clot is established showing similarities to final stages of coagulation cascade [9]. Fibrin clot, by holding the tissue, acts both as a homeostasis and as glue. Fibrin clot is considered to have a positive effect on anastomosis by forming a barrier, by bringing tissues end-to-end and facilitating them to remain that way while speeding up tissue recovery via the substances in its contents [9, 10].

A method is evidently needed to secure anastomosis and make it more reliable, even in the presence of intraabdominal infection avoiding surgeons to perform primary anastomosis in the first operation. Therefore, in our study, we aimed to expose the impact of performing fibrin glue on sutured colocolic anastomosis in the presence of experimental peritonitis on anastomosis safety. 

## 2. Material and Method

This research was conducted at the Marmara University, School of Medicine's Experimental Research and Animal Laboratory. Before starting the study, a project approval form was obtained from the Board of Test Animal Research Ethics, at the School of Medicine, Marmara University (42.2009.mar- 11.06.2009)^a^.

### 2.1. Method of the Study

Forty Norwegian Wistar Albino female rats, whose average age was 4 months and weight varied between 300 and 350 gr (with an average weight of 322 gr), were used in our study. The animals were separated into two equal groups of a control group and an experimental group. These two groups were then separated into two groups again as infected and clean abdomen. In the control group, 10 of the 20 rats were generated clean abdomen (Group 1), whereas the other 10 were created peritonitis (Group 3). Single layer colocolic anastomosis, only with end-to-end sutures, was performed after making full layer incision at the 5 cm distal level of the ileocecal valve. 

In the experimental group, 10 of the 20 rats were generated clean abdomen (Group 2), whereas the other 10 were created peritonitis (Group 4). One layer colocolic anastomosis, only with end-to-end sutures, was performed after making full layer incision at the 5 cm distal level of the ileocecal valve which was then applied with fibrin glue (Tisseel VH) over the anastomosis sufficient to cover it completely.

After sacrificing all rats on the 10th day postoperatively, tissue bursting pressure tests were initially performed on sample pieces of anastomotic lines. Afterwards, these tissues were sent to the biochemistry laboratory for measurement of hydroxyproline levels and to the pathology laboratory for measurement of fibroblastic activity and connective tissue levels. Histopathological examinations of subjects in all groups were conducted according to the Ehrlich-Hunt model [11]. 

Data collected via subject follow-up forms were transferred electronically by using the SPSS 11.5 program, and the same program was used in evaluating the data. In the statistical analysis, Mann-Whitney *U* test which is a nonparametric test was used to detect any significant differences among the control and experiment groups. *P* < 0.05 was considered statistically significant. 

### 2.2. Experiment Procedure

*Escherichia coli* (ATC 25227) strain necessary to generate peritonitis in subjects were obtained from the Microbiology Laboratory at the School of Medicine, Marmara University. Obtained strain has been incubated in the trypticase soy broth agar medium. Cultures were centrifuged for 5 minutes at a rotation speed of 2000. Formed pellets were homogenized via nephelometer forming Mc Farland 3. Obtained material was diluted via sterile saline as to contain 10^9^ *E. coli* (ATC 25227) per millimeter. 

Twelve hours before the operation, the animals were left without food, and they were administered a sugared water diet only. Twenty of these animals (Groups 3-4) were administered intraperitoneally 2 mL of *E. coli* (ATC 25227) isolate which was prepared in advance. Although there have been publications stating that 5 hours of waiting time would be sufficient, we waited for 12 hours to ensure widespread formation of peritonitis [12].

**Figure 1:**
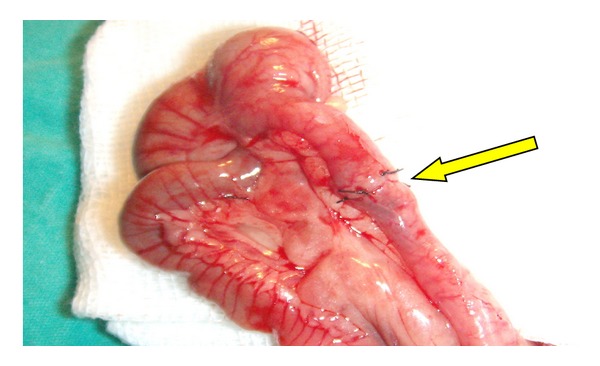
Colon anastomosis completed with suture.

**Figure 2:**
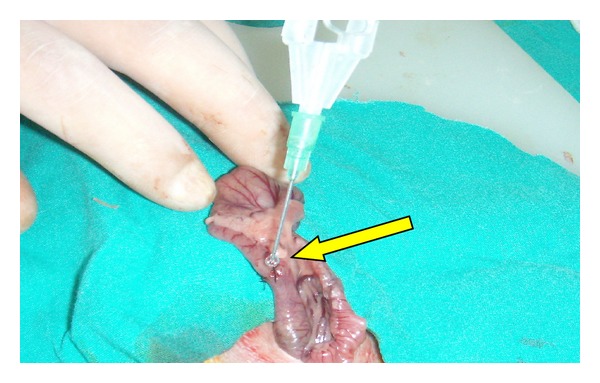
Application of fibrin glue on sutures.

**Figure 3:**
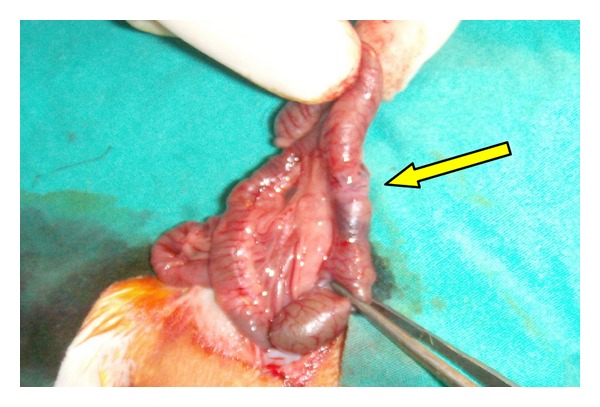
Appearance after application of fibrin glue.

General anesthesia was administered using 100 mg/kg Ketamine (Ketalar, Parke Davis and Co., Inc.) and Xylazin (Rompun, Bayer Ag, Leverkusen, Germany). After shaving the operation site, antisepsis was administered using povidone iodine. A full-thickness incision, at the 5 cm distal level of the ileocecal valve, was performed on proximal colon of all rats through a midline incision towards the abdomen by using a bistoury no. 15. The anastomosis on the 20 rats in the control group (Groups 1 and 3) was performed with one layer of Gambee sutures using round needles and 6/0 silk (Figure 1). Anastomosis performed on the 20 rats in the experiment group was also performed with one layer of Gambee sutures using round needles and 6/0 silk, and fibrin glue was applied over the anastomotic line so as to cover it completely (Figure 2). It has been waited for 3 minutes after the glue was applied (Figure 3). Subsequently, the abdomen was closed by suturing the fascia and skin separately using sharp needles and 4/0 silk. 

After the operations, the animals were placed into cages in groups of five and kept alive in a cycle of 12 hours night and 12 hours day. The cages were kept under normal ambient temperature and humidity conditions. All groups were given standard rat food and water. 

None of the animals have died before completion of the experiment. All subjects were performed relaparotomy under general anesthesia on the 10th day postoperatively. Anastomotic colonic segments operated beforehand were removed by resection so that 2 cm of healthy colon tissue remained on the proximal and distal parts of the anastomotic line. Subsequently, the rats were killed by exsanguinations.

The sample pieces obtained from the groups were initially subjected to anastomotic bursting pressure tests. Subsequently, half of the pieces containing the anastomotic line were stored in a physiological saline solution at a temperature of −22°C for biochemical measurement of hydroxyproline levels. The other half was stored in a 40% formaldehyde solution at a temperature of +4°C for histopathological examinations.

### 2.3. Anastomotic Bursting Pressure Measurement

Distal ends of all resected colon segments were firmly tied using 2/0 silk. A polyethylene catheter was placed into the lumen at the proximal end with the other end of the catheter connected to a transducer and an air pump. Thus, a setup necessary to view the intraluminal pressure in millimeters of mercury (mmHg) was obtained. The colonic segment was placed into a container filled with water, and air was pumped into the lumen at a speed of 4 mL/min. The first outlet of air from the anastomotic line was recorded as the anastomotic bursting pressure. 

### 2.4. Histopathological Examinations

The histopathological examinations were conducted by the same pathologist. The sample pieces were prepared in a paraffin block after which their thin cross-sections were dyed using “haematoxylin-eosin” (H-E) dye and examined under a light microscope. The images were recorded on a computer. Histopathological staging of the anastomotic line has been conducted according to the Ehrlich-Hunt model. Evaluation criteria in this model are amount of inflammatory cells, fibroblasts, neovascularization, and collagen (Table 1). 

### 2.5. Detection of Hydroxyproline Level in the Tissue

The quantity of hydroxyproline was detected using high performance liquid chromatography (HPLC). The tissues obtained were stored in a physiological saline solution at a temperature of −22°C. Subsequently, the materials were brought up to normal ambient temperature, and the study had started on them. In a laboratory environment, 1 gr tissue containing anastomotic line was homogenized in ether. Ether was extracted from the homogenized materials, and after buffering with phosphate, the study had started on them. Bio Lab Kit and Hitachi 911 device were used in measurement. The results were obtained in microgram/gram tissue (mcg/gram tissue).

## 3. Results

None of the animals have died neither during the operation nor during the time spent in obtaining the sample pieces. When subjects were killed on the 10th day postoperatively, a leakage has been detected in one of the anastomosis performed in Group 3, and an intraabdominal abscess had developed. Therefore, this subject was removed from the study.

### 3.1. Bursting Pressure

The average bursting pressure of groups was 232.5 ± 7,905 mmHg for Group 1, 269,5 ± 12,122 mmHg for Group 2, 201,1 ± 11,931 mmHg for Group 3, and 236,5. ± 20,554 mmHg for Group 4 (Table 2). Average bursting pressure of the subjects which were applied with fibrin glue on sutured colocolic anastomoses, both in clean abdomen and in the presence of peritonitis, was detected higher than the ones which were not applied with fibrin glue. Significant statistical differences have been detected among Groups 1 and 2 with clean abdomen and Groups 3 and 4 with peritonitis (*P* < 0.05).

### 3.2. Histopathological Evaluation

**Figure 4:**
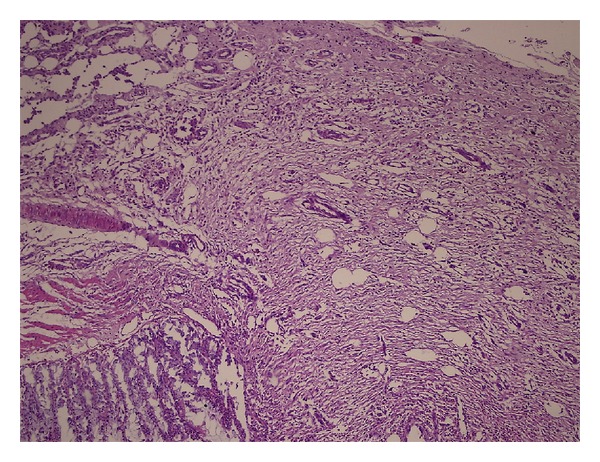
Intense fibroblastic activity, inflammatory cell infiltration, and vessel proliferation in Group 2 (HEx10).

**Figure 5:**
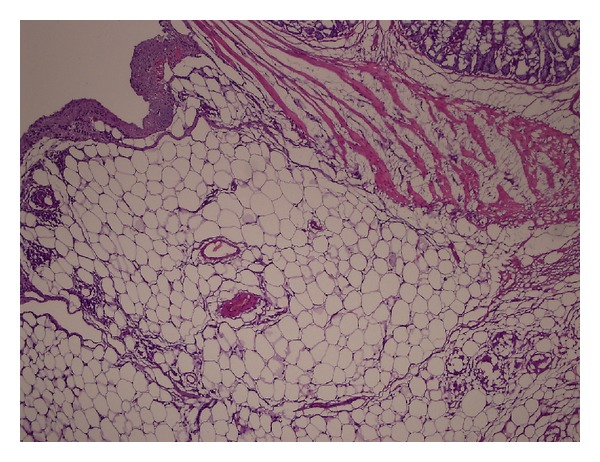
Minimal fibroblastic activity, inflammatory cell infiltration, and vessel proliferation in Group 3 (HEx10).

According to histopathological staging results based on the Ehrlich-Hunt model, the average values of the groups were detected as 13,5 ± 0,707 for Group 1, 14,6 ± 0,843 for Group 2, 9,1 ± 1,054 for Group 3, and 11,7 ± 1,159 for Group 4 (Table 2). To evaluate wound healing in the anastomotic line, according to the histopathological staging based on Ehrlich-Hunt model, all parameters (fibroblastic activity, inflammatory cell infiltration, neovascularization, and collagen) measured in Group 3 and Group 4 in the presence of peritonitis are in low degrees, whereas in Group 1 and Group 2, it has been detected that especially fibroblastic activity, inflammatory cell infiltration, and collagen bundles are intense. Sample microscopic images of the histopathological examination have been shown in Figures 4 and 5.

According to the histopathological evaluation, the average values of the subjects which were applied with fibrin glue on sutured colocolic anastomosis were detected higher than the ones that were not applied with fibrin glue. Significant statistical differences have been detected among Groups 1 and 2 with clean abdomen and Groups 3 and 4 with peritonitis (*P* < 0.05).

### 3.3. Level of Hydroxyproline

According to hydroxyproline levels (mcg/tissue) of the subjects, average values of the groups were detected as 196,4 ± 44,512 for Group 1, 266,2 ± 51,246 for Group 2, 108,8 ± 21,791 for Group 3, and 148,8 ± 24,593 for Group 4 (Table 2). Tissue hydroxyproline levels of the subjects which were applied with fibrin glue on sutured colocolic anastomoses, both in clean abdomen and in the presence of peritonitis, were detected higher than the ones that were not applied with fibrin glue. Significant statistical differences have been detected among Groups 1 and 2 with clean abdomen and Groups 3 and 4 with peritonitis (*P* < 0.05).

## 4. Discussion

Gastrointestinal system diseases in general surgery require education and skill on its own. It is difficult to ensure recovery without causing any complications in anastomoses performed in colorectal operations, especially harder in the presence of factors that threaten anastomosis safety. These factors are advanced age, infection, diabetes, loss of weight, emergency intervention, hypotension, long surgical intervention, surgeon's lack of experience, patient's state of nutrition, vascularity and tension of the anastomotic line and anastomosis technique. Among these, the only ones that are under the surgeon's control are vascularity, and tension of the anastomotic line and anastomosis technique [2]. In the published studies and series, the rates of anastomotic leakage vary between 1% and 30%. This rate may decrease down to 3%–6% with an experienced surgeon [13].

Primary anastomosis is avoided in the presence of contaminated abdomen (peritonitis), both in urgent and in elective surgical cases, but multistep procedures and administrating ostomy are preferred [3, 4]. The reasons for this are the deterioration of wound healing in a contaminated environment and high risk of anastomotic leakage, thus increasing morbidity and mortality. As a matter of fact, in our study, a leakage has been detected in Group 3 in an anastomosis in the presence of peritonitis. No findings indicating macroscopic anastomotic leakage are detected in the other groups.

Although still debated, publications are present indicating that only in the presence of peritonitis among all the risk factors, colonic resection and primary anastomosis may be performed with peritoneal hygiene and wide spectrum antibiotherapy [5, 14]. 

Bacteria which most frequently cause peritonitis among aerobes are Gram negative bacillus especially *E. coli* and among anaerobes are Gram negative bacteroides especially *Bacteroides fragilis* [12]. Therefore, we used *E. coli* in our study to generate experimental peritonitis. 

Many techniques and methods are being used to perform colonic anastomoses, and they all have the same objective: to generate the safest anastomosis. Therefore, surgical tissue adhesives have been tested for years. The most important examples of these adhesives are fibrin glue, bioglue, and cyanoacrylate. In the studies, it is reported that fibrin glue applied over anastomosis had been resorbed completely within 8–10 days, and its protective effect had ended [15, 16]. 

In the anastomotic bursting pressure tests, it has been stated that, starting on the 3rd day of the anastomosis, force to be applied increases gradually and reaches maximum on the 7th–10th days; at the same time, on the first 3 days, hydroxyproline concentration decreases by 40% in the anastomotic area, and approximately starting on the 5th day, it approaches normal level, whereas on the 10th–14th days, it rises above normal [17]. 

In this study, the first reason of performing relaparotomy on the subjects on the 10th day is to achieve maximum wound healing; the second is the removal of fibrin glue from the sample pieces by being totally resorbed and thus not affecting the results of hydroxyproline levels. 

In a study by Akgün et al., sutured colocolic anastomosis has been compared to the application of fibrin glue over the sutures. When subjects were compared in terms of anastomosis safety on the 72nd hour postoperatively, they have detected higher bursting pressures of the group that was applied with fibrin glue and have defended safer anastomosis [7]. The missing part of this study was having the evaluation only on the 3rd day postoperatively but not on the 8th–10th days postoperatively when wound healing was completed providing an increase in the anastomosis safety.

In a similar study by Kanellos et al., anastomosis safety had been inspected on the 8th day postoperatively, and similar data overlapping our study has been detected. They have stated that applying fibrin glue after sutured anastomosis had provided stronger anastomosis [18]. In another study by Kanellos et al., it was stated that fibrin glue, by wrapping anastomosis, protected healing from negative factors such as giving intraperitoneal 5-FU [15]. In our study, there was an infection which affected healing negatively, and even so, we detected that subjects' anastomoses that were applied with fibrin glue lasted longer.

Yılmaz et al., in their study to reveal the efficiency of fibrin glue in the presence of infection, had generated experimental peritonitis and applied fibrin glue on sutured anastomosis. In the results obtained on the 4th day postoperatively, they had stated that fibrin glue had increased anastomosis safety [19]. In a study by Li et al., it has been detected that subjects were killed on the 5th day postoperatively in the presence of peritonitis, and use of fibrin glue had increased wound healing [20]. When planning our study, we also aimed to observe the efficiency of fibrin glue in the peritonitis presence. We detected that in order to produce safer anastomosis, application of fibrin glue, whether infected or not, had increased anastomosis safety.

Although there have been many experiments on animals regarding the fibrin glue, the number of publications and studies regarding the use on patients is few. In a series of 42 patients by Romeo and Basile, gastrointestinal system anastomosis was performed, and fibrin glue (Tissucol) was applied over these anastomoses; yet, in none of the patients, development of anastomotic leakage was reported [21]. Huh et al., in a series of 223 rectum carcinoma patients, performed laparoscopic resection and stapling type of anastomosis and applied fibrin glue on anastomoses on randomly chosen 104 of the patients. In the postoperative followup, although the number of anastomotic leakage incidents was less in the group that was applied with fibrin glue, no significant differences were detected with the control group [14]. These studies may not reveal fibrin glue's efficiency in the presence of infection because all the patients had been prepared for the elective operation; therefore, peritoneum was not contaminated in any of them when the correct surgical technique was applied, and risky environment for anastomosis was not generated. 

On the other hand, there are also studies indicating that fibrin glue does not have a positive effect on anastomosis safety. In a study by Van der Vijver and Van Laarhoven to detect the effect of fibrin glue on early stage anastomosis safety, it was stated that there has been no significant effect over anastomosis on low risk cases, but it may be beneficial in the presence of cases leading to anastomotic leakage [16]. 

In a similar study by Giuratrabocchetta and Rinaldi, it was indicated that there has been no difference between the bursting pressures of groups that were applied and not applied with fibrin glue when evaluated on the 15th day postoperatively, and there has been no indication of fibrin glue decreasing the anastomotic leakage. Furthermore, intense tissue generation was reported in the group that was applied with fibrin glue [22].

In this experimental study, values of anastomotic bursting pressure, wound healing of tissues in histopathological examinations, and values of hydroxyproline levels of the subjects which were applied with fibrin glue over sutured colocolic anastomosis, both in clean abdomen and in the presence of peritonitis, were detected statistically significantly high.

In conclusion, we have come to conclusion that even in the presence of peritonitis which negatively affects wound healing, fibrin glue has a positive effect on anastomosis safety. Application of fibrin glue on risky sutured colocolic anastomosis will prevent both patients and surgeons from multistep procedures and administrating a stoma. Fibrin glue should not be used by itself but should be applied over sutured or stapled anastomoses. In this way, we are satisfied that stronger and more reliable anastomosis will be accomplished.

## Figures and Tables

**Table 1:** Model of Ehrlich-Hunt.

Stage	Inflammatory cells/fibroblasts/neovascularization/ collagen
1	Small amount but present in a scattered pattern
2	Small amount and present everywhere
3	High amount but present in a scattered pattern
4	High amount and present everywhere

**Table 2:** The Comparisons of bursting pressure and hydroxyproline levels and histopathological variables and scores within the groups.

Outcome measurements	Clean-glue (*n* = 10)	Clean-control (*n* = 10)	*P*	Peritonitis-glue (*n* = 10)	Peritonitis-control (*n* = 9)	*P*
Bursting pressure (mmHg)	269.5 ± 12.1	232.5 ± 7.9	<0.001	236.5 ± 20.6	201.1 ± 11.9	0.001

Hydroxyproline level (mcg/gram tissue)	266.2 ± 51.2	196.4 ± 44.5	0.002	148.8 ± 24.6	108.8 ± 21.8	0.001

Histopathological variables and score						
Total score	14.6 ± 0.8	13.5 ± 0.7	0.007	11.7 ± 1.2	9.1 ± 1.1	0.001
Inflammatory cell	3.8 ± 0.4	3.4 ± 0.5	0.075	2.2 ± 0.6	2.4 ± 0.5	0.576
Fibroblasts	3.7 ± 0.5	3.4 ± 0.5	0.189	3.1 ± 0.6	2.2 ± 0.4	0.007
Neovascularization	3.7 ± 0.5	3.4 ± 0.5	0.189	3.0 ± 0.7	2.2 ± 0.4	0.027
Collagen	3.4 ± 0.5	3.3 ± 0.5	0.648	3.8 ± 0.5	2.4 ± 0.5	0.004

(Data are presented in mean and standard deviations [±SD]).
